# Resensitization of cisplatin resistance ovarian cancer cells to cisplatin through pretreatment with low‐dose fraction radiation

**DOI:** 10.1002/cam4.2116

**Published:** 2019-04-02

**Authors:** Lili Zhao, Shihai Liu, Donghai Liang, Tao Jiang, Xiaoyan Yan, Shengnan Zhao, Yuanwei Liu, Wei Zhao, Hongsheng Yu

**Affiliations:** ^1^ Department of Oncology The Affiliated Hospital of Qingdao University Qingdao, Shandong Province China; ^2^ Department of Central Laboratory The Affiliated Hospital of Qingdao University Qingdao, Shandong Province China; ^3^ Department of Oncology Traditional Chinese medical hospital of Huangdao District Qingdao, Shandong Province China

**Keywords:** cisplatin resistance, FOXO3a, LDFRT, ovarian cancer

## Abstract

**Objective:**

Cisplatin is the first‐line chemotherapy for ovarian cancer. However, cisplatin resistance is severely affecting the treatment efficacy. FOXO3a has been reported to be involved in reversing chemotherapy resistance. However, whether low‐dose fraction radiation therapy (LDFRT) can reverse cisplatin resistance remains unclear. This study aimed to explore the effect of LDFRT on cisplatin resistance and its relation with FOXO3a expression in vitro.

**Methods:**

The toxicity of cisplatin on SKOV3/DDP cells was evaluated by CCK8 assay and cell apoptosis was measured by Annexin V‐FITC staining as well as Hoechst33342 staining. The expression of FOXO3a and other relative proteins was measured by western blot.

**Results:**

Our study found that LDFRT enhanced cisplatin‐induced apoptosis of SKOV3/DDP cells and promoted the expression of FOXO3a and pro‐apoptotic protein PUMA. In addition, overexpression of FOXO3a promoted PUMA activity and toxicity of cisplatin on SKOV3/DDP cells.

**Conclusion:**

LDFRT reverses cisplatin resistance of SKOV3/DDP cells possibly by upregulating the expression of FOXO3a and its downstream target PUMA, suggesting that LDFRT might be a potent chemosensitizer for the treatment of ovarian cancer.

## 1 INTRODUCTION

Ovarian cancer is the second leading diagnosed tumor in the female reproductive tract and metastasis has been observed in 80% of patients in the first diagnosis and more than 60% of metastasis occurs in the abdominopelvic cavity with a 5‐year survival rate less than 50%.^1^, ^2^, ^3^ Although cisplatin is beneficial in the initial treatment, after a period of time, cisplatin resistance occurs, which causes ovarian cancer recurrence and relapse in the abdominal cavity.^4^ Therefore, identifying a new useful way to reverse cisplatin resistance during the treatment of ovarian cancer is urgently required.

Radiation was discovered in the late 19th century and is widely used in the clinic including imaging diagnosis and cancers therapy. Ovarian cancer is sensitive to radiation and abdominal radiotherapy is an effective adjuvant radiotherapy for ovarian cancer, but the low tolerance of the up‐abdominal organs limits the application of convention radiation.^3^, ^5^

Low‐dose fraction radiation therapy (LDFRT), the total dose of low‐dose radiation divided into smaller doses called fractions, can improve the activity of the immune system and promote normal cells growth but inhibit cancer cells growth.^6^, ^7^, ^8^, ^9^ Clinical trials showed that LDFRT enhances the chemotherapeutic effect in human prostate cancer cells 10 and low‐dose abdominal radiation (60 cGy X 4 fractions) can act as a docetaxel chemosensitizer for recurrent ovarian cancer.^11^ However, whether LDFRT can reverse cisplatin resistance remains unclear.

*FOXO3a*, also known as forkhead boxO3, belongs to the family of forkhead transcription factors and plays a regulatory role in cells growth, differentiation, and apoptosis.^12^ Recently, some studies found that FOXO3a plays an important role in reversing chemoresistance and inhibiting tumor proliferation and development.^13^, ^14^, ^15^ Importantly, the accumulation of FOXO3 at laser‐induced damage was observed in cultured tumor cells treated by focused laser micro‐irradiation.^16^ PUMA, a downstream target of FOXO3a, was also implicated in the radiosensitivity of tumor cells.^17^, ^18^ In this study, we aimed to explore the effect of LDFRT on cisplatin resistance and its relation with FOXO3a and PUMA expression in vitro.

## 2 MATERIALS AND METHODS

### 2.1 Materials

The drug‐resistant ovarian cancer cell line, SKOV3/DDP, was bought from the Institute of Cancer Research, Chinese Academy of Medical Sciences (Beijing, China).^19^, ^20^ Cell counting kit‐8 (CCK‐8) and Annexin V‐FITC were purchased from Jiamay Biotech (The catalog numbers are AP1008 and LHK601‐100, respectively; China). Primary antibodies against FOXO3a or PUMA were purchased from Abcam (The catalog numbers are ab12162 and ab9643, respectively; USA). β‐actin antibody was purchased from Bioss (Catalog number: bs‐0061R; China). Plasmids GFP‐*Foxo3a* and GFP‐vector were purchased from Gene (Shanghai, China). Lipofectamine 2000 was purchased from Invitrogen (Catalog number: 11668019; USA).

### 2.2 Radiation treatment

Cells were cultivated in 1640 (HyClone ltd, China) medium supplemented with 10% fetal bovine serum (BI ltd, USA) and 1.25 µg/mL cisplatin to sustain drug resistance at 37℃ incubator with 5% CO_2_ and 95% O_2_. Cells were randomly divided into control group, low‐dose radiation (LDR) group, low‐dose fraction radiation (LDFRT) group and conventional group (CR). After cells reached a confluence of 40%‐50%, radiation treatment was applied. LDFRT group received two fractions of 0.5 gy per day (10:00 AM and 4:00 PM) for two days of continuous treatment.^21^ At the last radiation, the LDR group received 0.5 gy, CR group received 2 gy and the control group received no radiation. Twenty‐four hours after last radiation, all groups received cisplatin for another 24 hours followed by further analysis.

### 2.3 Cell proliferation/cytotoxicity assay

CCK‐8 is a more sensitive WST‐8‐based colorimetric assay than other tetrazolium salts such as MTT or MTS‐based assays in determining the cell viability regarding the cell proliferation and cytotoxicity. In cells, the tetrazolium salt, WST‐8, is reduced by dehydrogenases to generate a yellow color formazan dye, which is water soluble and directly proportional to the number of living cells.^22^ In our study, cells were digested by trypsin and replanted into 96‐well plates at a concentration of 5 × 10^3^ cells/100 µL followed by 24‐hour culture. Then, different concentrations of cisplatin (0, 1.25, 2.5, 5, 10, 20 µg/mL) were added into each well and incubated for 24 hours followed by addition of CCK8 reagent and incubation at 37°C. At the end time point, the optical density value at a wavelength of 490 nm was measured by a microplate reader (iMark^TM^, Bio‐Rad, USA). The viability in the cells without cisplatin treatment was set as 1.0, and this value was used to calculate the relative viability in cells treated with different concentrations of cisplatin. The GraphPad Prism software can easily fit a dose‐response curve to determine the IC50 (GraphPad Software, USA).

### 2.4 Apoptosis analysis

A total of 1 × 10^6^ cells were cultured overnight and collected by trypsin digestion. The cells were then washed with PBS followed by addition of Annexin V‐FITC and propidium iodide (PI) double staining and subsequent incubation at room temperature under dark for 15 minutes according to the manufacturer's protocol. Then, cell apoptosis was detected by a flow cytometer (BD Biosciences, USA).^23^

### 2.5 Western blot

Cells were washed with pre‐ice PBS and lysed with RIPA buffer for 30 minutes followed by centrifugation to collect the supernatant. The concentration of proteins was measured using the BCA protein assay kit. Proteins were separated by SDS‐PAGE at 100 V for 2 hours and wet transferred to the PVDF membranes at 350 mA for 1.5 hours. Then, the membrane was blocked with 5% skimmed milk at room temperature for 1 hour followed by incubation with primary antibodies (anti‐FOXO3a antibody, 1:2500; anti‐PUMA antibody, 1 µg/mL, and anti‐β‐actin antibody, 1:200） overnight at 4°C. After that, the membrane was washed with TBST three times and incubated with HRP‐conjugated second antibody (bs‐0295G‐HRP, 1:3000; Bioss, China) at room temperature for 2 hours. At last, the ECL reagent was added to visualize the proteins and band densities were determined by the ImageQuant TL software (GE Healthcare, USA).^24^ Every experiment was performed in triplicate.

### 2.6 Cell transfection

The cell transfection was conducted as previously described.^25^ Briefly, SKOV3/DPP cells were seeded into 24‐well plates. After 24‐h culture, the CMV‐MCS‐EGFP‐SV40‐FOXO3a plasmid or CMV‐MCS‐EGFP‐SV40 vector was transfected into cells using lipofectamine 2000 based on the manufacturer's protocol. Two days after transfection, the cells were examined under a fluorescence microscope (Leica, USA) and the overexpressed FOXO3a was confirmed using western blot.

### 2.7 Hoechst staining

We performed the Hoechst staining as previously stated.^26^ The SKOV3/DPP cells were seeded on glass coverslips (0.5 × 10^6^ cells/well) for 24 hours. Cells were then treated with cisplatin at the concentration of 5 µg/mL for 24 hours. Cells were fixed by 4% paraformaldehyde at room temperature for 10 minutes. Cells were then incubated in 1 μg/mL of Hoechst 33342 (Thermo Fisher Scientific, USA) for 1 hour, followed by washing twice with PBS. The coverslips were mounted by Fluoromount media (SouthernBiotech, USA) and examined using a fluorescent microscope (BX60; Olympus Optical Co., Tokyo, Japan).

### 2.8 Statistics analysis

All the experiments were repeated three times and data were processed by SPSS 20.0 software. The data were expressed as the mean ± standard deviation (SD).^27^ Student’s *t* test was performed for comparison of the differences between two independent groups and one‐way ANOVA was for the comparison of the differences between multiple groups. *P* < 0.05 was considered statistically significant.

## 3 RESULTS

### 3.1 The effects of cisplatin on SKOV3/DDP cells

**Figure 1:**
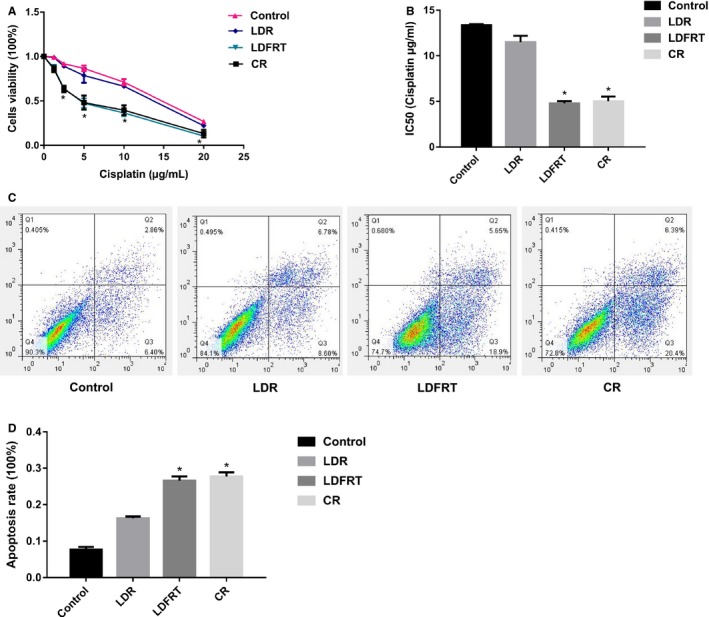
The effects of cisplatin on SKOV3/DDP cells. A, The toxicity of cisplatin on SKOV3/DDP cells. B, IC50 of cisplatin. C, Representative image showing the effects of cisplatin on the cell apoptosis of SKOV3/DDP cells. D, Quantification of the effects of cisplatin on the cell apoptosis of SKOV3/DDP cells. LDR, low‐dose radiation group; LDFRT, low‐dose fraction radiation group; CR, conventional group. Data were expressed as the mean ± SD. One‐way ANOVA was used for comparison of the differences between multiple groups. *P < 0.05 (n = 3) vs control group

To evaluate the toxicity of cisplatin on SKOV3/DDP cells, the CCK8 assay was performed. We found that, in all groups, the cisplatin affected the cell viability in a concentration‐dependent manner, with the higher concentration of cisplatin, the greater toxicity on SKOV3/DDP cells (Figure 1A). Furthermore, compared with the control group and the LDR group, the cell viability of SKOV3/DDP cells was much lower in the LDFRT group. Besides, the IC50 of cisplatin was much lower in the LDFRT group compared with the control group and LDR group (Figure 1B). Consistently, the cell percentage of cisplatin‐induced apoptotic SKOV3/DDP cells was much higher in the LDFRT group when comparing to the control group and LDR group (Figure 1C,D). Notably, similar results were obtained in the LDFRT and CR groups regarding the effects of cisplatin on SKOV3/DDP cells.

### 3.2 The expression of FOXO3a and relative proteins

**Figure 2:**
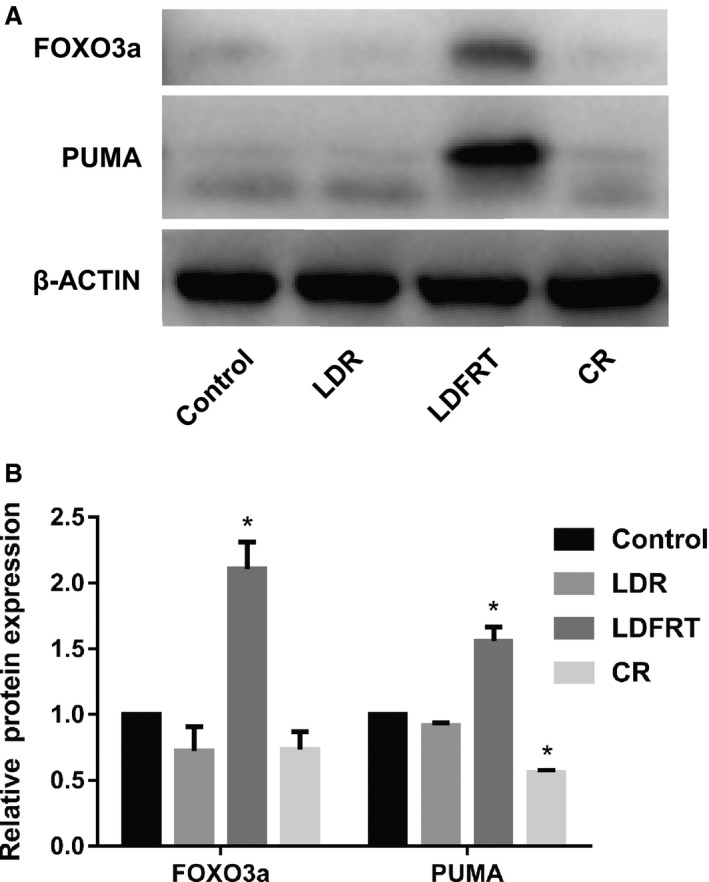
Western blot analysis of the expression of FOXO3a and PUMA. A, Representative western blot. B, Quantification of FOXO3a and PUMA protein levels. After 24‐h pre‐radiation, the expression of FOXO3a and PUMA was examined. Data were expressed as the mean ± SD. Student’s *t* test was performed for comparison of the differences between two independent groups. *P < 0.05 (n = 3) vs control group

After 24‐h pre‐radiation, the cells in the control, LDR, and CR groups had a lower expression of FOXO3a (Figure 2). However, LDFRT upregulated the expression of FOXO3a and its downstream target PUMA.

### 3.3 Overexpression of FOXO3a enhances the toxicity of cisplatin on SKOV3/DDP cells

**Figure 3:**
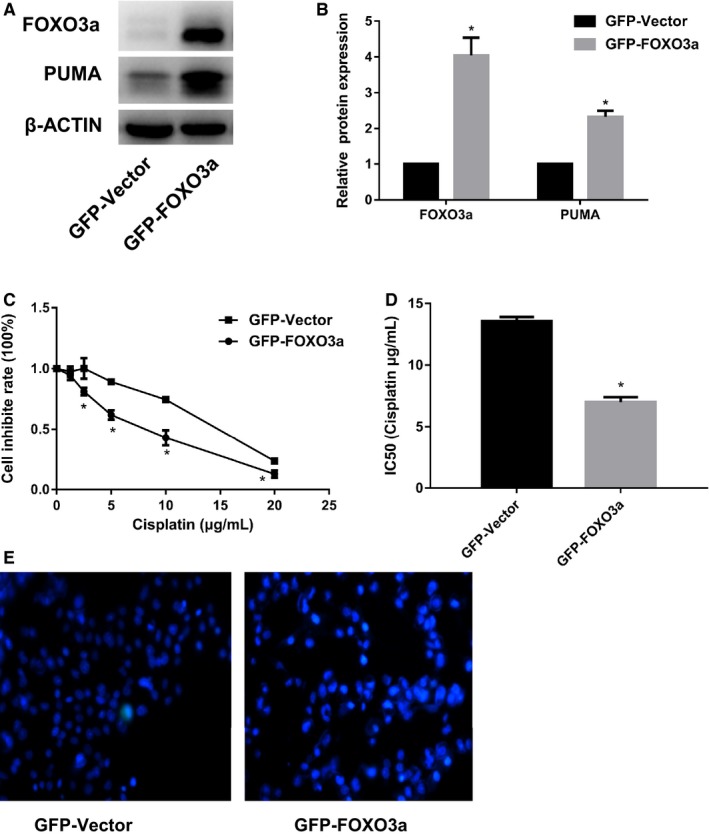
Effects of overexpression of FOXO3a on cisplatin‐induced toxicity and apoptosis of SKOV3/DDP cells. A, Representative western blot. The CMV‐MCS‐EGFP‐SV40‐FOXO3a plasmid was transfected into SKOV3/DDP cell followed by western blot analysis of the expression of FOXO3a and PUMA. B, Quantification of FOXO3a and PUMA protein levels. C, The toxicity of cisplatin on SKOV3/DDP cells. D, IC50 of cisplatin. E, Hoechst 33342 showing the cell apoptosis. After FOXO3a was overexpressed, cisplatin‐induced cell toxicity and cell apoptosis were measured by CCK8 assay and Hoechst33342 staining, respectively. Data were expressed as the mean ± SD. Student’s *t* test was performed for comparison of the differences between two independent groups. *P < 0.05 (n = 3) vs control group

To evaluate the role of FOXO3a in reversing cisplatin resistance, we transfected the SKOV3/DDP cells with the CMV‐MCS‐EGFP‐SV40‐FOXO3a plasmid to overexpress FOXO3a. As shown in Figure 3A,B, a significantly increased expression of FOXO3a and its downstream target PUMA were observed in SKOV3/DDP cells after transfection compared with the control group, indicating the successful transfection. We next assessed the effect of FOXO3a overexpression on cisplatin toxicity and apoptosis of SKOV3/DDP cells and found that overexpression FOXO3a increased the toxic effect of cisplatin (Figure 3C, D) and cisplatin‐induced cell apoptosis (Figure 3E) on SKOV3/DDP cells.

## 4 DISCUSSION

According to the USA cancer statistics, ovarian cancer is a high‐malignant female reproductive system tumor.^28^ There are no signs and symptoms in the early stages of ovarian cancer patients and some patients are already in the advanced stage at the time of being diagnosed.^1^ The standard therapeutic approach is cisplatin combined with surgical debulking for the advanced stage cancer.^28^ However, after initial treatment, cisplatin resistance is observed in some patients, which severely affects the treatment efficacy and outcome, leading to a lower 5‐year survival rate (<30%).^29^, ^30^ Therefore, discovering alternative approaches to overcome cisplatin resistance is extremely urgent.

Studies have shown that LDFRT can act as a chemosensitizer to reverse chemotherapy resistance,^11^, ^31^ as LDR can enhance immunity capacity, promote normal cells growth but inhibit cancer cells growth and induce radiation hypersensitivity.^6^, ^7^, ^8^, ^32^, ^33^, ^34^ Consistently, our study found that LDFRT enhanced the toxicity of cisplatin on SKOV3/DDP cells as well as cisplatin‐induced apoptosis of SKOV3/DDP cells.

**Figure 4:**
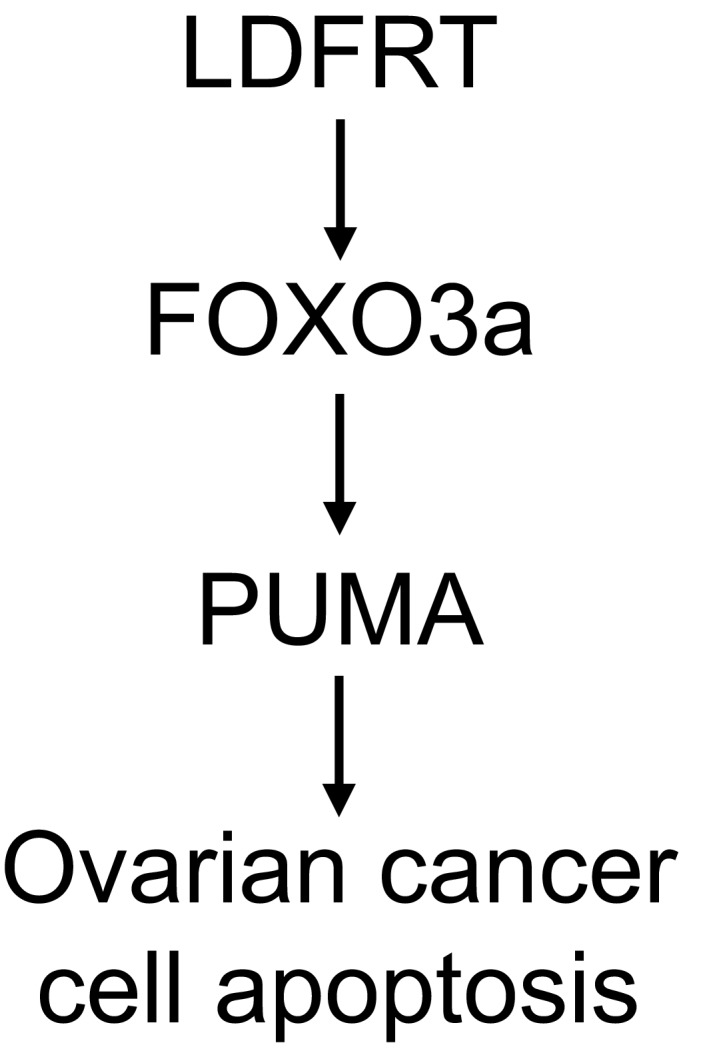
Diagram depicting the main players

As a tumor suppressor, FOXO3a in the nucleus is a conservative transcription factor that belongs to forkhead transcription factors and plays important roles in the regulation of cell differentiation, apoptosis, longevity, and metabolism.^35^, ^36^ The activity of FOXO3a depends on two patterns, posttranscription modification and subcellular localization. Phosphorylation is not only one of the posttranscriptions but also modifies the cellular localization of FOXO3a; the inactivity of FOXO3a was affected by phosphorylation of different sites.^39^ Although originally identified as a p53 downstream target,^40^, ^41^ PUMA expression was also regulated by FOXO3a in response to cytokine or growth factor deprivation. PUMA deficiency is known to protect cells from genotoxic stress that causes activation of p53. Additionally, cells lacking PUMA are also resistant to several p53‐independent death stimuli.^42^ Over the past three decades, studies have proved that many drug resistance cancer cells have a low expression of FOXO3a, which is a poor predictive factor. However, upregulation of FOXO3a can reverse cisplatin resistance.^38^, ^43^, ^44^ Consistent with these, in the present study, we found significantly lower expression of FOXO3a as well as its downstream target PUMA in SKOV3/DDP cells. However, LDFRT treatment significantly increased FOXO3a expression in SKOV3/DDP cells and reversed cisplatin resistance. In addition, overexpression of FOXO3a through transfection of the plasmid into SKOV3/DDP cells could also reverse cisplatin resistance together with increased expression of PUMA (Figure 4). Notably, PUMA expression is visible in the cells transfected with control vector, possibly due to the high basal level of PUMA in SKOV3/DDP cells.

In conclusion, our study shows that LDFRT promotes the expression of FOXO3a and its downstream target PUMA, as well as reverses cisplatin resistance, suggesting that low‐dose fraction radiation may be served as an effective complementary adjuvant radiotherapy in the treatment of ovarian cancer.

## CONFLICT OF INTEREST

All the authors have no conflict of interests to declare.
